# A Contribution to the Study of Uric Acid Diseases and Their Treatment*Read before the Mitchell District Medical Society, West Baden, Ind., 1897, and contributed exclusively to the *American Therapist*.

**Published:** 1898-03

**Authors:** Wm. Redin Kirk

**Affiliations:** Resident Physician Louisville City Hospital, 1891; Former Assistant to Chair of Gynecology in the Hospital College of Medicine, Visiting Gynecologist Louisville City Hospital, 1895; Assistant to the Chair of Materia Medica, Kentucky School of Medicine


### A CONTRIBUTION TO THE STUDY OF URIC ACID DISEASES AND THEIR TREATMENT.*

BY WM. REDIN KIRK, PH. G., M. D.

Resident Physician Louisville City Hospital, 1891; Former Assistant to Chair of Gynecology in the Hospital College of Medicine, Visiting Gynecologist Louisville City Hospital, 1895; Assistant to the Chair of Materia Medica, Kentucky School of Medicine.

The uric acid diathesis, embracing in its clinical aspect all the gradations from well-declared gout to those varied functional derangements depending as well upon faulty metabolism, is perhaps better known to-day than ever before, and perhaps as little understood as when Sir A. Garrod discovered the presence of uric acid in the blood and joints of gouty patients. There is no denying the fact that well-declared gout, associated with structural changes, and accompanied by pain and inflammation, is on the wane. Why this is so it is difficult to determine, but the fact also remains that in liéu of gout, in the aggregate form, we have a long list of ailments, which if not due to the presence of uric acid, are at least accompanied by it either in its free or combined state. Owing to the poverty of our nomenclature, no better term has been found than "goutiness," used by Ewart to express those varied functional disturbances and neuroses accompanied by uric acid and unasssociated with definite structural changes.

Whether gout is a local affection, or a constitutional disease with a local manifestation, does not concern us within the scope of this paper, further than to quote Ewart, who says the condition is one affecting the entire body and each cell within it, even the germ cells sharing in the alteration and transmitting it to the offspring. So general a change can only be due to a deep-seated process affecting metabolism itself. It would be interesting here to pause awhile, were the time allowed us, to speculate upon those changes which occur within the cell, and to study the force and influence of heredity upon the correlated diseases, gout, obesity and diabetes. For who could say, even though these diseases differ widely in their clinical appearance, that they may not yet be proven to have a common origin, and that common origin be found within the cell. For the origin of uric acid, and what is the starting point of disease, there are many theories familiar to

---

*Read before the Mitchell District Medical Society, West Baden, Ind., 1897, and contributed exclusively to the *American Therapist.*

us and unnecessary to be reviewed here. However, there are some of recent date so startling in originality and boldness that they demand our consideration. Among these the work of Horbaczewski, Kossel and others, as regards the derivation of uric acid from nuclein, and Haig's theory of uric acid and excessive vascular tension. Horbaczewski* found that uric acid might be derived from the decomposition of tissues. He found that it could be obtained in greatest quantity from the splenic pulp; but his researches were not confined to the spleen, he found it could be obtained from all the decomposing tissues in varying degrees. This he found also was not enough to explain the amount of uric acid in disease, and he believed that the leucocytes contained in the spleen and found in all the tissues in health, and in more abundance in some diseased conditions, must be the source of nuclein. Uric acid, he argued, would vary in proportion as there was a variation observed or induced in the aggregate number of leucocytes. He is borne out in this by the observations of R. C. Cabot (a guide to the Chemical Examination of the Blood, 1897), who found uric acid present in the blood after a meal of calf thymus or any food containing much nuclein. His theory of the derivation of uric acid finds confirmatory evidence in the recent treatment of goitre by thyroid extract, if Haig is correct in his views regarding raised arterial tension being due to the circulation of uncombined uric acid in the blood. I have observed this rise of arterial tension in patients taking thyroid extract, and have had to more than once discontinue the treatment or diminish the dose.

Sir A. Garrod* explains gout by assuming an excess of uric acid and laying the blame upon renal insufficiency and the subsequent deposit of urates in the joints. Haig† goes further and claims that uric acid is toxic in its soluble form, and that it is mechanically irritating when deposited in the joints. He claims that in its soluble form it expends its toxicity mostly upon the vascular and vaso-motor systems, thereby explaining a vast number of vague symptoms and conditions which have often been puzzling to the clinician.

A brother practitioner has kindly given me the result of some experiments upon himself, which substantiate Haig's theory:

He has been a great sufferer from migraine, and testing his urine before, during and after the attacks, he found uric acid present when the headache was worst, and absent before and after each attack. The attacks were invariably accompanied by raised arterial tension. Since attention has been called by

*J. Horbaczewski, "Beitrage zur Kenntniss der Bilddung der Harnasure uud der Xanthinosen," *Monatsheft fur Chemie*, x, pp. 221—226.

*Lecons sur les Maladies du Foie des Rains, page 320, 1877.

†Alexander Haig, Uric Acid as a Factor in the Causation of Diseases, etc., Second Edition, 1894!

Haig to the effect of uric acid upon the vascular and vaso-motor systems, clinicians have observed any number of functional derangements and disease conditions traceable to it as a cause. Asthma, various skin affections, tonsilitis, pharyngitis, bronchitis, migraine, neuralgias, etc., and numerous neuroses traceable to disturbed vaso-motor equilibrium, not to mention the results following the presence of gravel, stone or uratic deposits in the joints and tissues.

It is possible, also, that the renal insufficiency observed in gout and rheumatism by Garrod and others, and contributing as a cause in determining an attack of gout by preventing the elimination of uric acid, may be at first functionally caused by uric acid as such before its combination into insoluble compound, and not caused by the cell retention of the acid as bio-chemists would have us believe. It matters not what theory we may accept, the therapeutical indications remain the same whether treated by one method or another. These are the prevention of the formation of uric acid by dietetic, prophylactic and hygienic measures.

I shall not consume your time with remarks on the prevention of the formation of uric acid, but will consider only the question of how best to deal with the condition when it is found. No other diathesis has so thoroughly exhausted therapy as has this one. We have vacillated and veered from one extreme to the other with varying success. The inorganic alkaline treatment has been tried and proven to be of only temporary value. The lithia salts, so much lauded as uric acids solvents, I believe to be almost worthless. Sir E. Roberts has proven by numerous experiments that when they do combine with uric acid they form insoluble compounds, very difficult of elimination. It has remained, at least in my hands, for the organic alkaline salts to accomplish the solution and elimination of the urates. And among these the best results have been obtained from piperazine alone, or in combination with phenocol, as best administered in piperazine water.

The following case, substantiating Haig's theory, is submitted for your consideration:

Mrs. W., age forty, a highly cultured and refined woman, of neurotic temperament, a chronic sufferer from migraine; had intermittent attacks of severe frontal headache for the last six years. Examination of the nose revealed no obstruction or catarrhal condition; health otherwise good; family history revealed long line of gouty, rheumatic and asthmatic ancestry. During the attacks, which came on suddenly, there was intense pain, raised arterial tension, which, after the first day, subsided until the pulse was almost imperceptible. If the attack was very severe there was loss of consciousness. She ate little or nothing during these attacks, and the renal secretion was diminished, high in color and filled with brick-dust deposits, and examination showed an increase of uric acid and urates during the attack, and an absence of these substances in the interval of good health.

In view of the family history and her physical condition, she was placed upon piperazine, which very materially lessened the amount of uric acid and the severity of the pain. It was also noticed that the arterial tension subsided in a very marked degree. Later I put her on piperazine water with gratifying results. The almost absence of pain I attributed to the combined action of piperazine and phenocol. She was, of course, restricted in diet, and given the usual directions as to hygiene.

This case alone bears out the conclusions of Haig and others, and to my mind establishes the value of organic against inorganic alkaline salts. I omitted to say, in her case I did not try the usual remedies in such cases, as she had previously run the gamut of the entire pharmacopeia.

In conclusion I may add, that I have been fortunate enough to relieve two cases of chronic asthma traced to uric acid as a cause, by the use of piperazine, which I consider the best of all the organic uric acid solvents.—*American Therapist.*

